# Robust radiogenomics approach to the identification of *EGFR* mutations among patients with NSCLC from three different countries using topologically invariant Betti numbers

**DOI:** 10.1371/journal.pone.0244354

**Published:** 2021-01-11

**Authors:** Kenta Ninomiya, Hidetaka Arimura, Wai Yee Chan, Kentaro Tanaka, Shinichi Mizuno, Nadia Fareeda Muhammad Gowdh, Nur Adura Yaakup, Chong-Kin Liam, Chee-Shee Chai, Kwan Hoong Ng

**Affiliations:** 1 Division of Medical Quantum Science, Department of Health Sciences, Graduate School of Medical Sciences, Kyushu University, Fukuoka, Japan; 2 Faculty of Medical Sciences, Division of Medical Quantum Science, Department of Health Sciences, Kyushu University, Fukuoka, Japan; 3 Faculty of Medicine, Department of Biomedical Imaging, University of Malaya, Kuala Lumpur, Malaysia; 4 Department of Respiratory Medicine, Kyushu University Hospital, Fukuoka, Japan; 5 Division of Medical Sciences and Technology, Department of Health Sciences, Graduate School of Medical Sciences, Kyushu University, Fukuoka, Japan; 6 Faculty of Medicine, Department of Medicine, University of Malaya, Kuala Lumpur, Malaysia; 7 Faculty of Medicine and Health Science, Department of Medicine, University Malaysia Sarawak, Kota Samarahan, Sarawak, Malaysia; Universita degli Studi di Perugia, ITALY

## Abstract

**Objectives:**

To propose a novel robust radiogenomics approach to the identification of epidermal growth factor receptor (*EGFR*) mutations among patients with non-small cell lung cancer (NSCLC) using Betti numbers (BNs).

**Materials and methods:**

Contrast enhanced computed tomography (CT) images of 194 multi-racial NSCLC patients (79 *EGFR* mutants and 115 wildtypes) were collected from three different countries using 5 manufacturers’ scanners with a variety of scanning parameters. Ninety-nine cases obtained from the University of Malaya Medical Centre (UMMC) in Malaysia were used for training and validation procedures. Forty-one cases collected from the Kyushu University Hospital (KUH) in Japan and fifty-four cases obtained from The Cancer Imaging Archive (TCIA) in America were used for a test procedure. Radiomic features were obtained from BN maps, which represent topologically invariant heterogeneous characteristics of lung cancer on CT images, by applying histogram- and texture-based feature computations. A BN-based signature was determined using support vector machine (SVM) models with the best combination of features that maximized a robustness index (RI) which defined a higher total area under receiver operating characteristics curves (AUCs) and lower difference of AUCs between the training and the validation. The SVM model was built using the signature and optimized in a five-fold cross validation. The BN-based model was compared to conventional original image (OI)- and wavelet-decomposition (WD)-based models with respect to the RI between the validation and the test.

**Results:**

The BN-based model showed a higher RI of 1.51 compared with the models based on the OI (RI: 1.33) and the WD (RI: 1.29).

**Conclusion:**

The proposed model showed higher robustness than the conventional models in the identification of *EGFR* mutations among NSCLC patients. The results suggested the robustness of the BN-based approach against variations in image scanner/scanning parameters.

## 1. Introduction

Lung cancer is the leading cause of cancer-related deaths worldwide [1]. Approximately 85% of lung cancer lesions are of the non-small cell lung cancer (NSCLC) subtype [2]. The 5-year survival rates for stages I, II, III, and IV NSCLC are approximately 80, 57, 25, and 5%, respectively [3]. The treatment of patients with late-stage epidermal growth factor receptor (*EGFR*) sensitizing mutations positive NSCLC using tyrosine kinase inhibitors (TKIs) is exemplified as precision medicine which takes into account the individual variability in the genes, environment, and lifestyle of each patient.

**Fig 1:**
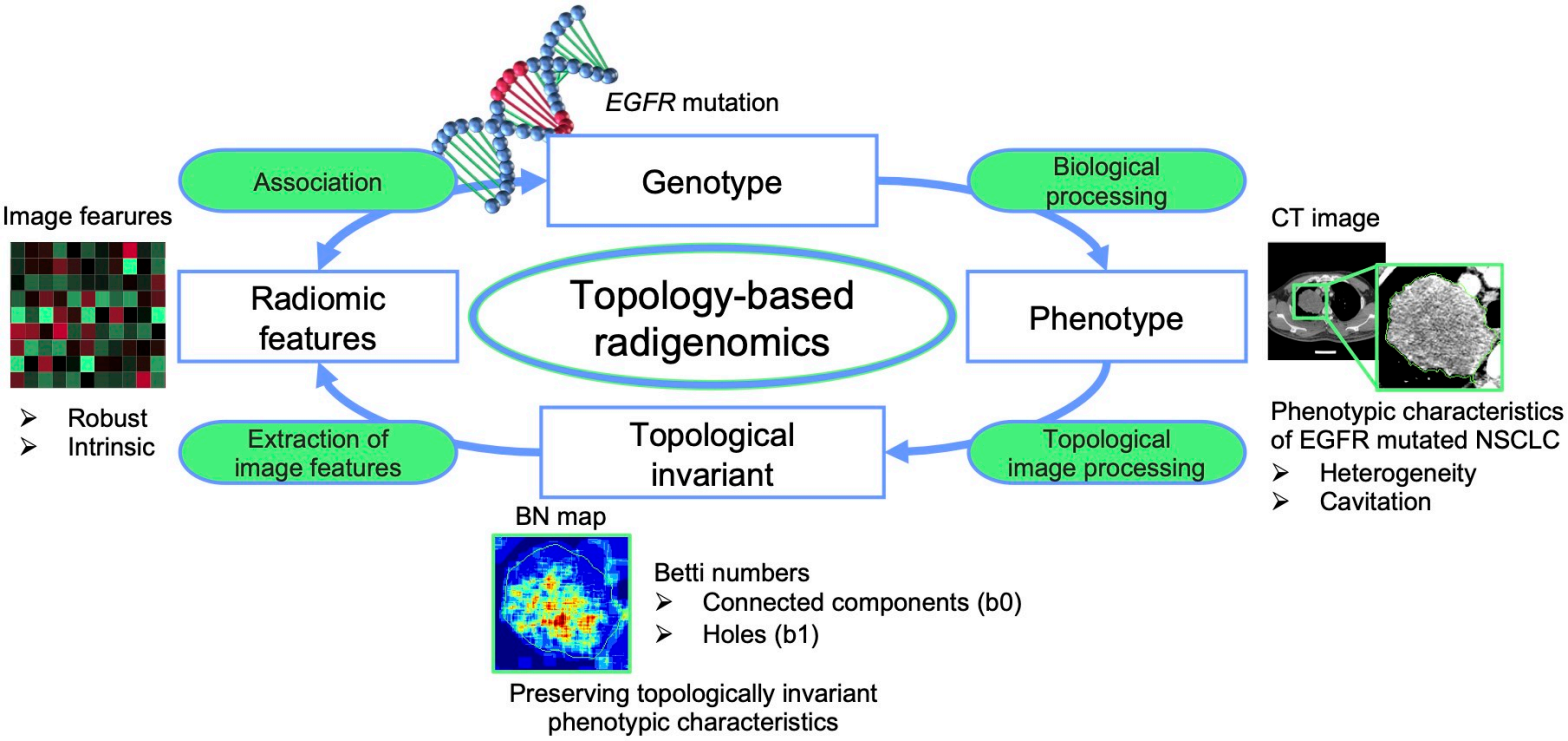
Assumption and concept of the present study. The assumption of this study is that epidermal growth factor receptor (*EGFR*) mutation in non-small cell lung cancer (NSCLC) leads remarkable phenotypes appearing on computed tomography (CT) images and that could be characterized by using Betti numbers (BN) derived from topology theory.

In a rapidly developing field of radiomics, researchers have been investigating the associations between medical images and patients’ prognostic information (including the *EGFR* mutation status) in a non-invasive manner under the assumption that particular somatic mutations of cancer lead remarkable phenotypes appearing on the medical images (Fig 1) [4–7]. Previous studies demonstrated underlying associations between the *EGFR* mutation status and intra-tumor heterogeneity on computed tomography (CT) images quantified using conventional radiomic features such as shape-, original image (OI)-, wavelet-decomposition (WD)-, and deep learning-based features [8–10]. However, past studies have not investigated the feasibility of their model using datasets with wide variety of imaging parameters or patient populations [8,9], although it was reported that conventional radiomic features can struggle to extract intrinsic image features that are robust to variations in CT scanner or scanning parameters [11,12].

Betti numbers (BNs), which are topological invariant in the homology, has been applied to quantify tumor traits in several medical images such as CT, magnetic resonance, and pathological images [13–17]. The topologically invariants in the BNs indicates unchangeable property of objects under continuous deformation [18]. In two-dimensional images, two types of BNs can be defined, that are zero- and one-dimensional BNs representing the number of connected components (b0) and holes (b1), respectively. In fact, several studies reported that *EGFR* mutated NSCLC appeared to have intra-tumor heterogeneity on contrast enhanced (CE) CT images [19] and hazy areas in tumor regions which cause low-intensity holes on the CT images [20]. Meanwhile, in our previous study, we found that b0 and b1 could robustly characterize the intra-tumor heterogeneities and the low-intensity holes associated with prognoses of lung cancer patients, although those features were extracted from the CT images acquired under several scanner/scanning parameters [5]. Therefore, in the present study, we assumed that the BN-based image features could also successfully quantify the intra-tumor heterogeneity and the low-intensity holes related with the *EGFR* mutations in the robust manner.

Here, we present our work on the robust radiogenomics approach based on topologically invariant BNs to the identification of the *EGFR* mutations among patients with NSCLC in three databases acquired from three different countries using 5 manufacturers’ scanners with a variety of scanning parameters.

## 2. Methods

### 2.1 Clinical cases

The study protocol was approved by the institutional review boards of Kyushu University Hospital (KUH) and University of Malaya Medical Centre (UMMC). Table 1 and S1–S3 Tables summarize the demographic/clinical characteristics and significant differences between patients with the *EGFR* mutant and wildtype tumors in the datasets obtained from UMMC (Malaysia), KUH (Japan) and The Cancer Imaging Archive (TCIA) (America) [21]. The CE CT images of 194 NSCLC patients (79 *EGFR* mutants and 115 wildtypes) were analyzed. Ninety-nine cases obtained from UMMC were used for training and validation procedures. Forty-one cases collected from KUH and fifty-four cases obtained from TCIA were used for a test procedure. The case numbers selected from TCIA database were listed in S4 Table [21]. These CT images were acquired using several scanners with a variety of scanning parameters (Tables 2 and 3) in which there were statistically significant differences in slice thickness and in-plane pixel size between the training (validation) and the test datasets (Mann–Whitney *U* test, p < 0.05) (Table 3). The matrix sizes after reconstruction were 512 × 512 × 46–750 (median, 346), 512 × 512 × 38–415 (median, 114) and 512 × 512 × 115–636 (median, 250) for the datasets obtained from UMMC, KUH and TCIA, respectively.

**Table 1:** Distributions and significant differences in demographic/clinical characteristics between patients with sensitizing epidermal growth factor receptor (*EGFR*) mutants and wildtypes in datasets obtained from University of Malaya Medical Centre, Kyushu University Hospital and The Cancer Imaging Archive.

	*EGFR* mutant	*EGFR* wildtype	p value (testing method)
Total number of cases	79	115	
Age (y, min-max (median))	28–87 (66)	27–89 (68)	0.24 (Mann-Whitney U-test)
Sex			3.79 × 10^−6^ (Chi-squared test)
Male	33	87	
Female	46	28	
Stage			8.67 × 10^−3^ (Mann-Whitney U-test)
I	13	24	
II	6	13	
III	5	25	
IV	55	53	
Smoking status			1.73 × 10^−9^ (Mann-Whitney U-test)
Non-smoker	54	26	
Former-smoker	16	50	
Current-smoker	9	39	
Ethnicity			
Asian	70	70	2.62 × 10^−4^ (Chi-squared test)
Caucasian	8	43	
Hispanic/Latino	1	1	
Native Hawaiian/Pacific Islander	0	1	

**Table 2:** Image acquisition parameters in datasets obtained from University of Malaya Medical Centre (UMMC), Kyushu University Hospital (KUH) and The Cancer Imaging Archive (TCIA).

Dataset	Manufacturer	No. of cases	Model	No. of cases	Reconstruction Kernel	No. of cases
UMMC	SIEMENS	92	Definition	39	B20f	71
			Definition AS+	17	B30f	5
			Sensation 16	18	B31f	15
			SOMATOM Definition AS	1	D20f	1
			SOMATOM Definition AS+	17		
	GE	5	LightSpeed16	5	Standard	5
	Philips	1	Ingenuity CT	1	B	1
	TOSHIBA	1	Aquilion ONE	1	FC08	1
KUH	TOSHIBA	22	Aquilion	1	EC	2
			Aquilion 64_3	4	FC13	1
			Aquilion 64_4	10	FC53	1
			Aquilion ONE	5	FC81	1
			ID_station	2	FC86	16
					LARGE	1
	Philips	16	Brilliance 64	4	B	2
			iCT256	10	C	1
			Ingenuity CT	1	YB	1
			PHILIPS-95201	1	YC	12
	SIEMENS	2	SOMATOM Definition AS+	2	B60f	1
					B70f	1
	CANON	1	Robust	1	9	1
TCIA	GE	48	Discovery CT750 HD	33	BONE	1
			LightSpeed VCT	1	BONEPLUS	9
			Unavailable	14	LUNG	34
					STANDARD	4
	SIEMENS	5	Unavailable	5	B45f	3
					B50f	1
					B70f	1
	TOSHIBA	1	Unavailable	1	FC52	1

**Table 3:** Comparison of voxel sizes and slice thicknesses in datasets obtained from University of Malaya Medical Centre (UMMC), Kyushu University Hospital (KUH) and The Cancer Imaging Archive (TCIA).

		UMMC	KUH	TCIA
Voxel size (mm)		0.53–0.98 (0.73)	0.37–0.74 (0.39)	0.60–0.98 (0.79)
pvalue (Mann-Whitney U-test)	v.s. KUH	1.07 × 10^−17^	–	–
	v.s. TCIA	1.49 × 10^−7^	4.62 × 10^−16^	–
Slice thickness (mm)		0.50–5.00 (0.75)	1.00–5.00 (2.00)	0.63–2.50 (1.25)
pvalue (Mann-Whitney U-test)	v.s. KUH	1.70 × 10^−20^	–	–
	v.s. TCIA	4.71 × 10^−18^	3.31 × 10^−10^	–

The *EGFR* mutation status was examined among lung tumor specimens using the QIAGEN EGFR RGQ PCR Kit (QIAGEN, Manchester Ltd, UK), Cobas^®^ EGFR Mutation Test (Cobas^®^, Roche Molecular System Inc., USA), PNAClamp^™^ EGFR Mutation Detection Kit (PANAGEN, Daejon, Korea), or the PNA-LNA PCR clamp (LSI Medience, Japan). The tumor specimens were obtained via image-guided biopsy, endobronchial biopsy, or percutaneous Tru-Cut^®^ needle biopsy as clinically indicated. For the detailed description of TCIA dataset, see ref. [22].

Solid tumor regions were defined for each patient as regions of interest (ROIs) to be analyzed using ITK-SNAP [23] and a 3D slicer [24]. All segmentations were performed and/or verified by a radiologist or respiratory physician with more than eight years of experience (WYC and KT).

Anisotropic CT images and ROIs were transformed into isotropic images with an isovoxel size of 0.77 mm (mode pixel size in the training dataset), using cubic and shape-based interpolations [25], respectively. In the present study, an axial plane of each CT image, which contained the maximum axial plane area of the ROIs, was selected for the calculation of radiomic features [26,27]. Since annotations of the ROIs are not created as a routine work in the clinical practice, we considered our algorithm should work with minimal labor. Aside from that, several past studies reported that two-dimensional radiomic features based on the CT images with maximum solid tumor areas showed comparable performance to the three-dimensional features in characterization of tumors [26,27]. Therefore, the two-dimensional feature extraction algorithms were applied in the present study.

A multiple-segmentation dataset, which was used to evaluate the robustness of the signatures against inter-observer variability, consisted of CT images of patients with NSCLC (n = 30) from the Quantitative Imaging Network multisite collection of lung CT data, with nodule segmentations from TCIA (detailed in ref. [5]) [28–30]. The ROI in each image was independently segmented by three different institutions: Columbia University Medical Center, Stanford University, and Moffitt Cancer Center/University of South Florida. Each institution performed segmentation using their own custom segmentation algorithms under three different sets of initial conditions. These configurations resulted in nine segmentations of each tumor, for a total of 270 segmentations. The case numbers for constructing the multiple-segmentation dataset were listed in S5 Table.

### 2.2 Betti number maps computation

**Fig 2:**
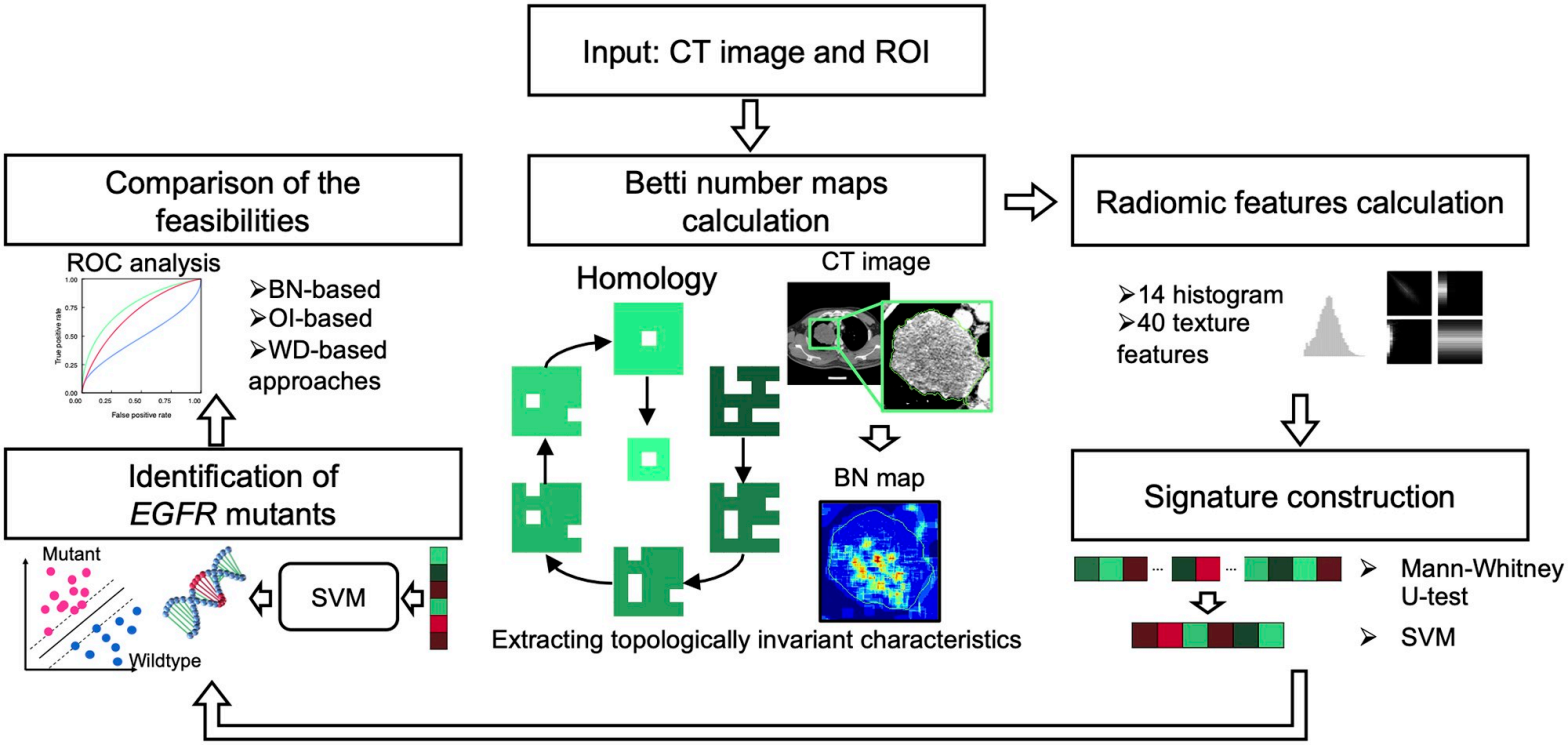
Overall workflow for the present study. CT: Computed tomography, ROI: Region of interest, BN: Betti number, SVM: Support vector machine, *EGFR*: Epidermal growth factor receptor, OI: Original image, WD: Wavelet-decomposition.

**Fig 3:**
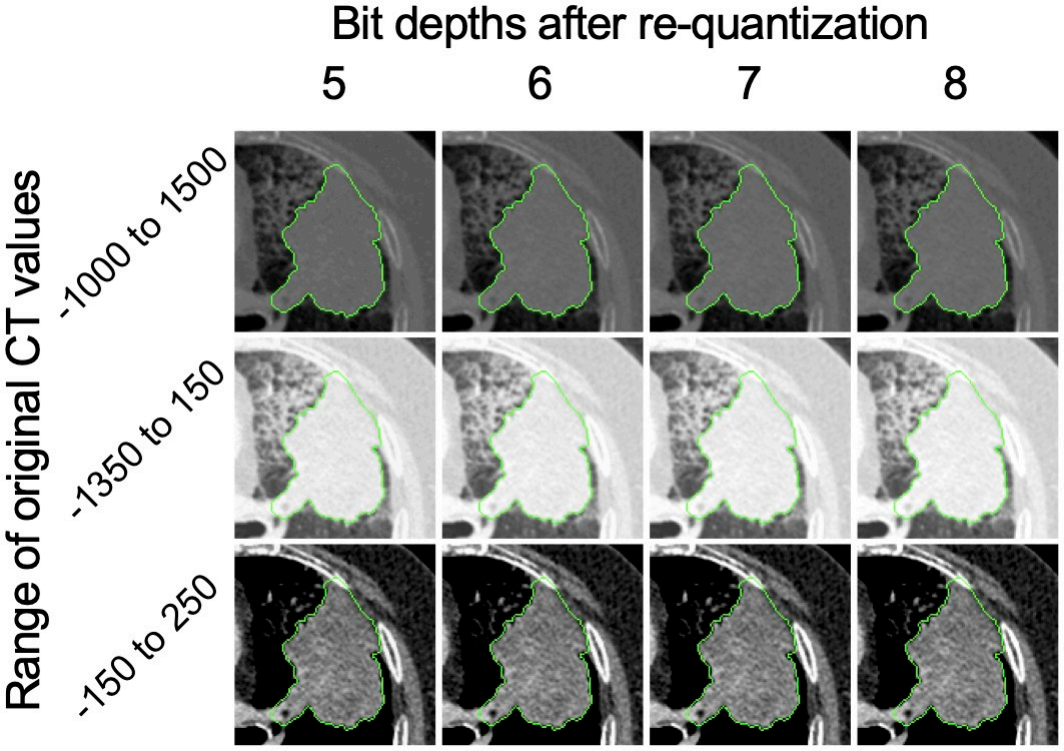
*q*-bit images generated from the computed tomography (CT) images using four ranges of Hounsfield units (HU) of CT images (-1000 to 1500 HU, -1350 to 150 HU [lung range window], and -150 to 250 HU [mediastinal range window]) with four bit-depths after re-quantization (5, 6, 7, and 8 bits).

**Fig 4:**
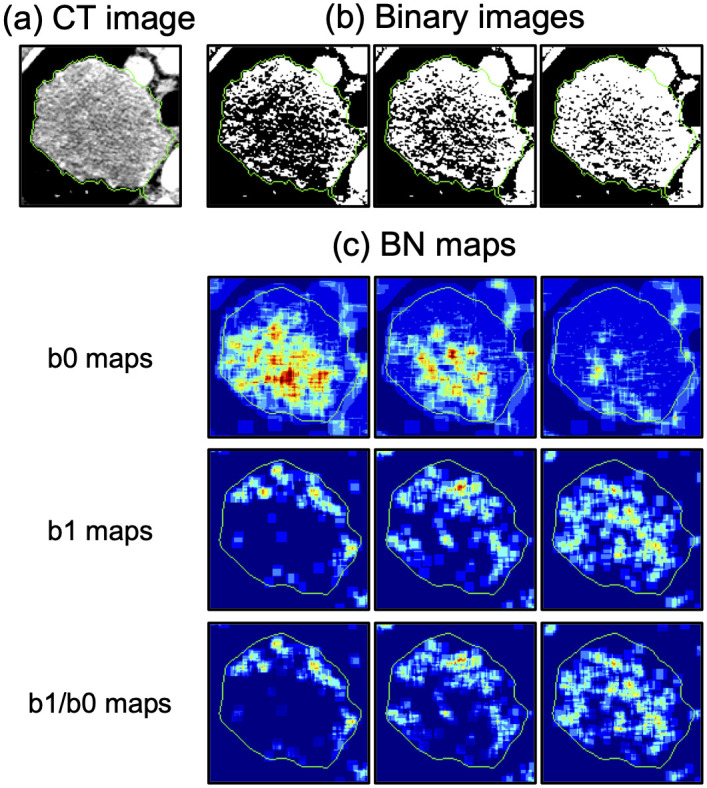
Representative images of (a) computed tomography (CT) images, (b) binary images, and (c) Betti number (BN) maps.

Fig 2 shows an overall workflow of the present study. The BN maps were computed from a *q*-bit CT image (Fig 3) by counting the number of connected components (b0), holes (b1), and holes per connected component (b1/b0) through thresholding (Fig 4) and a convolutional computation procedure [5]. A total of 2^*q*^ × 3 BN maps (b0, b1, and b1/b0 maps) were calculated from binary images which were derived from the *q*-bit CT images by thresholding the images with values ranging from 0 to 2^*q*^ − 1. The *q*-bit CT images were obtained by re-quantizing the original CT images into *q* bits. The optimal re-quantization level was explored among twelve types of *q*-bit CT images generated from the CT images using three ranges of Hounsfield units (HU) of the CT images (-1000 to 1500 HU [5], -1350 to 150 HU [lung range window], and -150 to 250 HU [mediastinal range window]) with four bit-depths after re-quantization (5, 6, 7, and 8 bits) (Fig 3). The kernel sizes and shifting pixels in the calculation of BNs using the convolutional computation were optimized from four kernels (5, 7, 9, and 11 pixels squared) and five shifting pixels (1, 2, 3, 4, and 5 pixels). The detailed algorithms used in the computation of the BN maps are shown in ref. [5].

### 2.3 Extraction of radiomic features

Fifty-four features were computed from the BN maps within the ROI by applying fourteen histogram- and forty texture-based (nine from a gray level co-occurrence matrix (GLCM), thirteen from a gray level run-length matrix (GLRLM), thirteen from a gray level size-zone matrix (GLSZM), and five from a neighborhood gray-tone difference matrix (NGTDM)) feature calculations. The total number of features could be calculated as 2^*q*^ × 3 × 54 (the number of BN maps × the number of image features). Therefore, a total of 41,472, 20,736, 10,368, and 5,184 BN features were extracted from the BN maps based on the eight-, seven-, six-, and five-bit CT images, respectively. S6 Table shows a list of the features computed in the present study (see Supporting information).

### 2.4 Construction of radiomic signatures

Radiomic signatures were determined using the best combination of features that maximized a robustness index (RI) evaluating the feasibility and robustness of the support vector machine (SVM) models with a set of features in the identification of the *EGFR* mutants. The best combination was searched among representative features, which were the features showing statistically significant differences (Mann–Whitney *U* test, p < 0.05) between median feature values for the *EGFR* mutants and wildtypes. Based on a review paper published by Chalkidou et al. [31], it was not necessary to correct the threshold values for statistical significance (p < 0.05) in the present study, as independent tests were performed. The number of constituent features in the signature was determined from one to four using the methodology put forth by Vallières et al. [32] to search for the best combination of the features by maximizing the RI for training and validation defined as:

$$RI=\frac{AUC_{train}-AUC_{valid}}{1+|AUC_{train}+AUC_{valid}|},$$

where *AUC*_*train*_ and *AUC*_*valid*_ indicate the AUCs for the identification of the *EGFR* mutants in the training and the validation procedures, respectively. These AUCs were obtained in the five-fold cross validation using the SVMs, which were constructed using a Gaussian kernel with a soft margin parameter *C* of 1 and gamma of 1/N (N: the number of features) [33]. Since we applied the five-fold cross validation, the *AUC*_*train*_ was calculated by averaging the AUCs obtained in the five training procedures.

### 2.5 Construction of classifiers for the identification of the *EGFR* mutants

The SVMs were used to build the identification models of the *EGFR* mutants using the BN-based signature. The SVM was implemented with linear, Gaussian, and sigmoid kernels and several soft margin parameters *C* ranging from 0.1 to 10 with an interval of 0.3. For the Gaussian and the sigmoid kernels, gamma was also optimized using the values from 0.1 to 10 with an interval of 0.3. These parameters were optimized by maximizing the RI for training and validation using a grid search strategy.

### 2.6 Construction of conventional models

OI- and WD-based models were built to compare the feasibility of the BN-based model. The OI-based features were extracted from original CT images using 54 feature calculation methods described in subsection 2.3. A total of 216 WD features were obtained by applying the 54 feature calculation methods to four WD images. These WD images were obtained from *q*-bit CT images by applying either a low-pass filter (L) or a high-pass filter (H), which was derived from a Coiflet 1 mother wavelet, along the x and y axes. Therefore, the number of WD images resulted in four [LL, LH, HL and HH (First character L or H represents a low- or a high-pass filter applied along the x axis and second character represents the same for y axis)] The radiomic signatures and the SVMs for OI- and WD-based models were constructed in the same way as the BN-based models described in subsections 2.3–2.5.

### 2.7 Evaluation of the identification performance and robustness

The robustness of radiomic signatures against inter-planner variabilities of the ROIs was evaluated using mean intra-class correlation coefficients (ICCs) calculated in the multiple-segmentation dataset (refer to subsection 2.1) for the constituent features in the BN- and the WD-based signatures. Their robustness was evaluated using the following criteria for the ICCs [34]: poor robustness, ICC < 0.5; moderate robustness, 0.5 ≤ ICC < 0.75; good robustness, 0.75 ≤ ICC < 0.9; and excellent robustness, ICC ≥ 0.9.

The BN-based model was compared to the conventional OI- and WD-based models in terms of accuracy and robustness of *EGFR* mutant identification. The models were evaluated using the RI based on the AUCs from the validation and the test procedures. A higher value of the RI determined the best model which could accurately and/or stably identify the *EGFR* mutants in both the validation and the test procedures.

The 95% confidence intervals (CIs) of the AUCs were estimated via bootstrapping (2000 times).

### 2.8 Statistical analysis

In the demographic/clinical characteristics, Mann-Whitney U-tests were applied to assess significant differences of age, stage and smoking status between the *EGFR* mutant and the wildtype. On the other hand, chi-squared tests were applied for sex and ethnicity. Stages from I-IV were used as numerical values to apply the Mann-Whitney U-test. In comparison of smoking status, numerical values were assigned for each category; non-smoker: 1, former-smoker: 2, current-smoker: 3.

Mann-Whitney U-test was applied to assess statistically significant differences of the CT scanning parameters of slice thickness and in-plane pixel size among the UMMC, the KUH and the TCIA datasets.

Statistical differences among the AUCs obtained from the BN- and the OI- or the WD-based approaches were evaluated using a Delong’s test (significance threshold; p < 0.05) [35]. All analyses were performed using R-4.0.3 (available at http://www.r-project.org/).

## 3. Results

Table 4 shows a summary of mean ICCs, the AUCs with CIs, accuracies, sensitivities, specificities and the RIs from the BN-, the OI- and the WD-based models to the identification of the *EGFR* mutants.

**Table 4:** Summary of mean intra-class correlation coefficients (ICCs), areas under the receiver operating characteristic curves (AUCs) with 95% confidence intervals (CIs), accuracies, sensitivities, specificities and robustness indices from Betti number (BN)-, original image (OI)-, and wavelet decomposition (WD)-based models to the identification of epidermal growth factor receptor mutants.

		BN	OI	WD
	Mean ICC	0.84	0.75	0.070
Validation	AUC (95% CI)	0.86 (0.78–0.93)	0.69 (0.58–0.79)	0.65 (0.54–0.76)
	Accuracy	0.84	0.67	0.62
	Sensitivity	0.78	0.58	0.75
	Specificity	0.88	0.73	0.53
Test	AUC (95% CI)	0.77 (0.67–0.86)	0.54 (0.42–0.66)	0.71 (0.59–0.81)
	Accuracy	0.71	0.66	0.74
	Sensitivity	0.72	0.38	0.69
	Specificity	0.70	0.86	0.77
	Robustness index	1.51	1.33	1.29

The mean ICCs for the BN-, the OI- and the WD-based signatures were 0.84 (good robustness), 0.75 (good robustness) and 0.070 (poor robustness), respectively. Four (b0_GLCM_Energy_45, b1/b0_GLSZM_ZSN_104, b1_GLCM_SumAverage_122, b0_GLRLM_LRLGE_97), three (GLRLM_SRLGE, GLSZM_LGZE, GLSZM_SZLGE) and one (GLSZM_LGZE_LL) features were selected in the signatures for the BN, the OI and the WD, respectively. Digits in the range of 0–255 following the names of the BN features correspond to the threshold values for obtaining BN maps. The optimal parameters of the BN map computations were the kernel size of 7, the shifting pixel of 1, and the 8-bit CT image obtained using the mediastinal window (-150 to 250 HU). The optimal parameters for the BN-based SVM model were the Gaussian kernel with the soft margin parameter *C* of 0.4 and the gamma of 0.4. The optimal SVM parameters for the OI-based model were the Gaussian kernel, the soft margin parameter *C* of 7.3 and the gamma of 2.5. The optimal parameters for the WD-based model were the 7-bit CT image obtained using the lung window (-1350 to 150 HU) and the SVM using the Gaussian kernel with the soft margin parameter *C* of 7.3 and the gamma of 2.5.

**Fig 5:**
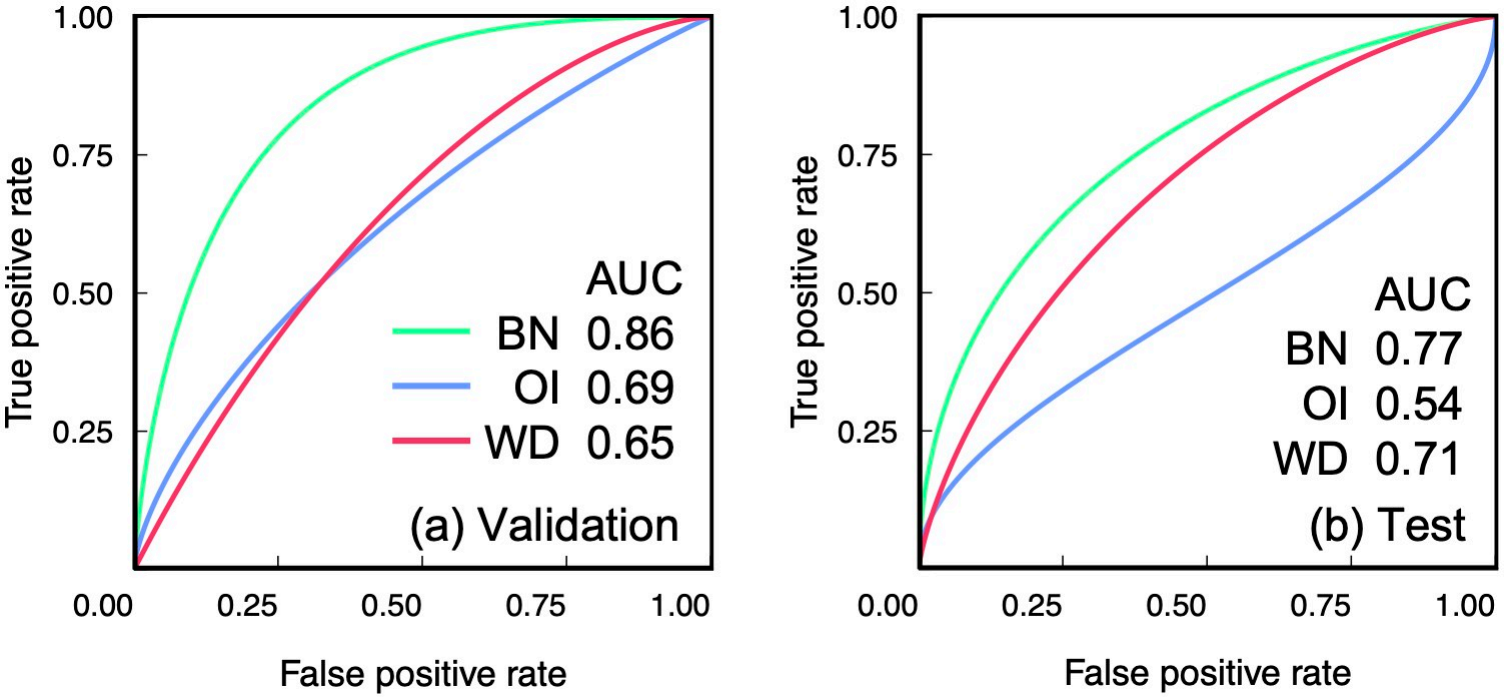
Receiver operating characteristic (ROC) curves for identification of epidermal growth factor receptor mutants using Betti number (BN)-, original image (OI)- and wavelet decomposition (WD)-based models with area under the ROC curves (AUC) in (a) the validation and (b) the test procedures.

Fig 5 shows the ROC curves for the identification of the *EGFR* mutants using the BN-, the OI- and the WD -based models. In the BN-based model, the AUCs for the validation and the test were 0.86 (CI: 0.78–0.93) and 0.77 (CI: 0.67–0.86), respectively. On the other hand, the AUCs in the OI-based model for the validation and the test were 0.69 (0.58–0.79) and 0.54 (0.42–0.66), respectively. Further, the AUCs in the WD-based model for the validation and the test were 0.65 (CI: 0.54–0.76) and 0.71 (CI: 0.59–0.81), respectively (Table 4). The p values between two AUCs obtained between the BN- and the OI-based models in the validation and the test procedures were 1.4 × 10^−4^ and 5.8 × 10^−3^ (Delong’s test), respectively. Besides, the p values between two AUCs obtained between the BN- and the WD-based models in the validation and the test procedures were 3.1 × 10^−3^ and 0.29 (Delong’s test), respectively. The BN-based model showed a higher RI of 1.51 compared with the OI-based model (RI: 1.33) or the WD-based model (RI: 1.29).

**Fig 6:**
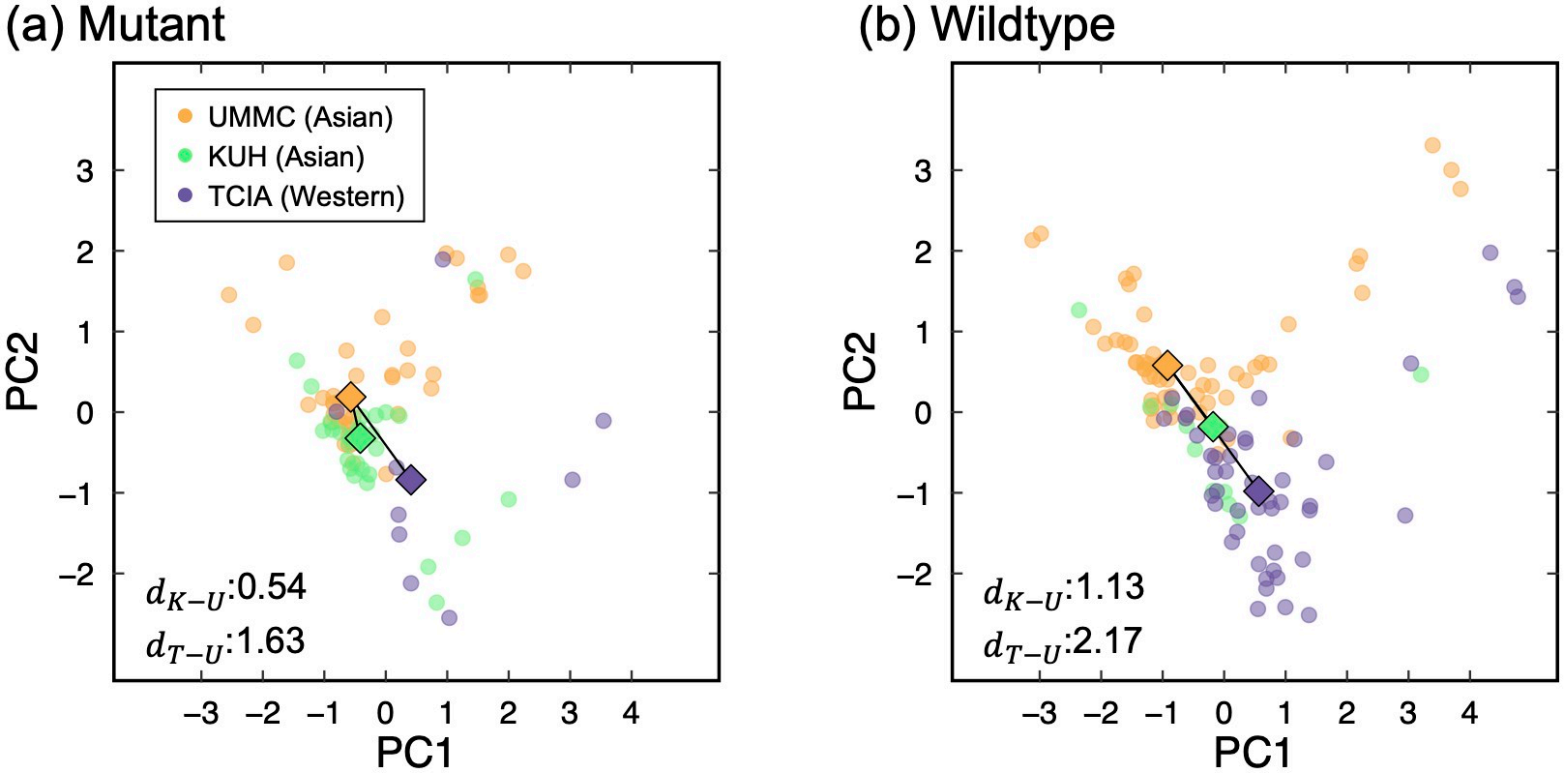
Distributions of signatures for (a) epidermal growth factor receptor mutants and (b) wildtypes among datasets from three countries. Euclidean distances between medians of the signatures from University of Malaya Medical Centre (UMMC) and The Cancer Imaging Archive (TCIA) (*d*_*T*−*U*_) were larger than that from UMMC and Kyushu University Hospital (KUH) (*d*_*K*−*U*_). PC1 and PC2 represent first and second principal components, respectively, calculated among constituent features in the signature. The medians were represented by square-shaped plots.

In the test procedure using the BN-based model, the AUCs for KUH and TCIA were 0.76 (CI: 0.60–0.90) and 0.61 (CI: 0.41–0.79), respectively. Fig 6 shows distributions of the signatures for the *EGFR* mutants and the wildtypes among the datasets from three countries. Euclidean distances of medians of the BN-based signature, which was normalized using z-score, between UMMC and KUH for the mutants and the wildtypes were 0.54 and 1.13, respectively. On the other hand, the distances between UMMC and TCIA for the mutants and the wildtypes were 1.63 and 2.17, respectively.

Table 5 summarizes the identification performance of the present and previous studies that performed the validation and the test of the models [8,9]. Although our dataset consisted of patients from three different countries, the BN-based approach showed similar RIs compared with previous studies that used domestic databases.

**Table 5:** Comparison of the results of areas under the receiver operating characteristic curves (AUCs) and robustness indices obtained from the present study and previous studies.

Author (year)	Number of cases	Feature type	Datasets	Validation and test datasets	Origin of datasets (country)	AUC		Robustness index
						Validation	Test	
Present study	194	Betti number	Training 99 Testing 95	Independent datasets from three institutions	Malaysia, Japan, America	0.86	0.77	1.57
		Original image (OI)				0.69	0.54	1.33
		Wavelet decomposition (WD)				0.65	0.71	1.29
Wang (2019) ref. [8]	844	Deep learning	Training 603 Testing 241	Independent datasets from two institutions	China	0.85	0.81	1.60
Yang (2019) ref. [9]	467	shape + OI +WD	Training 306 Testing 161	Random split datasets from one institution	China	0.83	0.79	1.56

## 4. Discussion

Almost 50% of Asian lung cancer patients harbor tumors with TKI-sensitizing *EGFR* mutation positive NSCLC [36,37], whereas prevalence of the *EGFR* mutations in Western lung cancer patients was about 15% [38]. Prevalence rate of the *EGFR* mutations in our datasets was similar to these general proportions (Asian [UMMC and KUH]: 50.00% [*EGFR* mutatns:wildtypes = 50:100], Western [TCIA]: 16.67% [*EGFR* mutatns:wildtypes = 9:45]). The small number of the *EGFR* mutations in the Western patients could be attributable to the lower AUC of 0.61 in the test for the TCIA dataset as compared with the AUC of 0.76 for the KUH dataset. Aside from that, we also found that Euclidean distances of medians of the BN-based signature between UMMC and TCIA were larger than that from UMMC and KUH (Fig 6). The image features between Asian and Western could potentially differ from each other. These differences in the image features also lessened the AUCs in the test for the TCIA dataset.

The BN-based model showed stable identification performance in both the validation and test datasets, although the CT images were acquired using various scanning parameters (Tables 2 and 3). This result suggested that the BN-based signature was robust to differences in the scanning parameters. The b0, b1 and b1/b0 maps evaluated the number of the connected components composed of high-intensity pixels, the number of holes caused by low-intensity pixels and the density of low-intensity holes, respectively. These calculation procedures might be suitable for preserving the characteristics of the *EGFR* mutants and dismissing the effects of differences in the quality of the CT images. As a result, the proposed BN-based model could perform as well as the model developed by Wang et al. [8] that used four times more cases as in the present study.

The presence of ground-glass opacity (GGO) has been recognized as one of representative characteristics associated with presence of the *EGFR* mutations [19,20]. The GGO is defined as a hazy area of increased attenuation of the lung with preservation of bronchial and vascular margins which cause low-intensity holes on the CT images [20]. In addition, intra-tumor heterogeneity on CE CT images has also been reported to have the association with the *EGFR* mutations in NSCLC [19]. Those findings were only explored in a qualitative manner. Our results had similar tendency to those of the qualitative analysis [19]. In the present study, we developed the robust model to quantify their heterogeneity using the topologically invariant BNs.

The RI obtained in the previous radiomics study was higher than that obtained using our conventional approaches [9]. Aside from the experiments using the OI- and the WD-based features, we also assessed the feasibility of shape-based features extracted using two- and three-dimensional calculations provided by pyradiomics package in Python. However, there were no representative features which were significantly associated the *EGFR* mutation status. In the previous study conducted using conventional radiomic analysis (Table 5), dataset was composed of larger number of cases from a single institution. Therefore, there might be smaller variability in the image qualities.

A BN- and clinical factor (BC)-based SVM model was also constructed using the signature of the BN and clinical factors of sex and smoking status because they were significantly associated with the *EGFR* mutation status in the training dataset (S1 Table). The optimal SVM parameters for the BN-based model was used for the BC-based model. The identification performance of the BC-based model was similar to that of the BN-based model. In the BC-based model, the AUCs for the validation and the test were 0.81 (CI: 0.72–0.89) and 0.77 (CI: 0.66–0.87), respectively.

Worldwide application of the model for the identification of the *EGFR* mutants requires the investigation of the feasibilities of the model with respect to multiple databases across the world. However, past studies mentioned above [8,9] have not conducted evaluation of the model using patient data from different countries. That fact motivated us to conduct the present study with an international database consisted of patients with NSCLC from three different countries.

The *EGFR* mutations promote cellular proliferation, differentiation, and migration of NSCLC [39]. EGFR-TKIs such as gefitinib, erlotinib, afatinib, and osimertinib for patients with advanced *EGFR* mutant NSCLC are the standards of care for first-line treatment. These agents confer significantly longer median progression-free survival compared to standard platinum-based doublet chemotherapy [40]. Therefore, the *EGFR* mutations in patients with NSCLC should be accurately identified for the selection of optimal treatments in precision medicine [41]. Currently, the *EGFR* mutations among patients with NSCLC can be identified using different platforms such as direct sequencing, real-time polymerase chain reaction, or immunohistochemistry on tissue specimens obtained via image-guided invasive needle biopsies, bronchoscopy biopsies, or surgical resection [42]. However, these invasive procedures are associated with discomfort and potentially serious complications such as pneumothorax, bleeding, airway trauma, infection, and rarely death [43–45]. In addition, invasive procedures may not be feasible in some patients due to physical unfitness, co-morbidities, and reluctance [46]. Therefore, non-invasive approaches to assessing the *EGFR* mutation status are preferable and may provide more patients with the opportunity to undergo targeted treatment with the EGFR-TKIs. The present study could facilitate a non-invasive and reliable approach for the detection of the *EGFR* mutations.

The present study had four limitations. First, the number of cases was small although the proposed model showed potential to be robust and feasible in the identification of *EGFR* mutants. Past studies used at least two times as much number of cases as this study [8,9]. If we add more patient data in the analysis, the model’s performance could be further improved and confident. Second, three-dimensional computations of the BNs have not been performed. The application of three-dimensional BN features may lead more accurate identification of the *EGFR* mutants by reflecting volumetric information of heterogeneous lung tumors. Third, we did not assess the impact of the time between contrast injection and the CT scan. Yang et al. reported that some conventional radiomic features showed variability depending on the time at which the CT scan was obtained after contrast injection [47]. Since the BN-based features were computed from the BN maps obtained through thresholding to the CE CT images, the timing of the CT scans after injection may affect the quantitative values of the features. Finally, we only focused on identifying the *EGFR* mutations from the others (wildtypes). It would be necessary to have other mutation groups such as KRAS mutations and ALK fusions.

## Conclusions

The proposed model based on the topologically invariant BN outperformed the conventional identification models. The results of the present study suggested the robustness of the BN-based approach against variations in scanner/scanning parameters of three different countries. Therefore, the BN-based approach showed potential in the non-invasive identification of the *EGFR* mutations and assist physicians to tailor more effective treatment strategies for NSCLC patients.

## Supporting information

S1 Table Distributions and significant differences in demographic/clinical characteristics between patients with sensitizing epidermal growth factor receptor (*EGFR*) mutants and wildtypes in a dataset obtained from University of Malaya Medical Centre. (DOCX)

S2 Table Distributions and significant differences in demographic/clinical characteristics between patients with sensitizing epidermal growth factor receptor (*EGFR*) mutants and wildtypes in a dataset obtained from Kyushu University Hospital. (DOCX)

S3 Table Distributions and significant differences in demographic/clinical characteristics between patients with sensitizing epidermal growth factor receptor (*EGFR*) mutants and wildtypes in a dataset obtained from The Cancer Imaging Archive. (DOCX)

S4 Table Case numbers obtained from The Cancer Imaging Archive for constructing a test dataset. (DOCX)

S5 Table Case numbers selected from The Cancer Imaging Archive for constructing a multi segmentation dataset. (DOCX)

S6 Table Radiomic features with the feature types. (DOCX)
