# Correction: Robust radiogenomics approach to the identification of *EGFR* mutations among patients with NSCLC from three different countries using topologically invariant Betti numbers

**DOI:** 10.1371/journal.pone.0316496

**Published:** 2024-12-20

**Authors:** Kenta Ninomiya, Hidetaka Arimura, Wai Yee Chan, Kentaro Tanaka, Shinichi Mizuno, Nadia Fareeda Muhammad Gowdh, Nur Adura Yaakup, Chong-Kin Liam, Chee-Shee Chai, Kwan Hoong Ng

In **2.4 Construction of radiomic signatures of** Methods Section, there is an error in the definition of the robustness index (RI). The correct RI is defined as:

$$RI=\frac{{AUC}_{train}+{AUC}_{valid}}{1+|{AUC}_{train}-{AUC}_{valid}|}$$

where *AUC_train_* and *AUC_valid_* indicate the area under receiver operating characteristics curves (AUCs) for the identification of the *EGFR* mutants in the training and the validation procedures, respectively.
