# Supplementary material for: Robust radiogenomics approach to the identification of EGFR mutations among patients with NSCLC from three different countries using topologically invariant Betti numbers
Source: PLoS One. 2021 Jan 11;16(1):e0244354. doi: 10.1371/journal.pone.0244354 (PMC7799813; doi:10.1371/journal.pone.0244354)
Supplement: S1 Table — (DOCX) [file pone.0244354.s001.docx]

**S1 Table. Distributions and significant differences in demographic/clinical characteristics between patients with sensitizing epidermal growth factor receptor (*EGFR*) mutants and wildtypes in a dataset obtained from University of Malaya Medical Centre.**

|  | *EGFR* mutant | *EGFR* wildtype | p value (testing method) |
| --- | --- | --- | --- |
| Total number of cases | 40 | 59 |  |
| Age (y, min-max (median)) | 32-83 (63) | 27-89 (66) | 0.36 (Mann-Whitney U-test) |
| Sex |  |  | 3.94 × 10^−3^  (Chi-squared test) |
| Male | 16 | 42 |  |
| Female | 24 | 17 |  |
| Stage |  |  | 0.06  (Mann-Whitney U-test) |
| I | 1 | 2 |  |
| II | 0 | 1 |  |
| III | 2 | 10 |  |
| IV | 37 | 46 |  |
| Volume (cm^3^, min-max (median)) | 0.53-411.60 (31.42) | 1.30-946.51 (43.04) | 0.34 (Mann-Whitney U-test) |
| Smoking status |  |  | 1.44 × 10^−4^  (Mann-Whitney U-test) |
| Non-smoker | 31 | 20 |  |
| Former-smoker | 3 | 19 |  |
| Current-smoker | 6 | 20 |  |
| Ethnicity |  |  |  |
| Asian | 40 | 59 | 1.00 (Chi-squared test) |
