# Supplementary material for: Robust radiogenomics approach to the identification of EGFR mutations among patients with NSCLC from three different countries using topologically invariant Betti numbers
Source: PLoS One. 2021 Jan 11;16(1):e0244354. doi: 10.1371/journal.pone.0244354 (PMC7799813; doi:10.1371/journal.pone.0244354)
Supplement: S2 Table — (DOCX) [file pone.0244354.s002.docx]

**S2 Table. Distributions and significant differences in demographic/clinical characteristics between patients with sensitizing epidermal growth factor receptor (*EGFR*) mutants and wildtypes in a dataset obtained from Kyushu University Hospital.**

|  | *EGFR* mutant | *EGFR* wildtype | p value (testing method) |
| --- | --- | --- | --- |
| Total number of cases | 30 | 11 |  |
| Age (y, min-max (median)) | 28-80 (68) | 51-84 (66) | 0.95 (Mann-Whitney U-test) |
| Sex |  |  | 6.41 × 10^−3^  (Chi-squared test) |
| Male | 11 | 10 |  |
| Female | 19 | 1 |  |
| Stage |  |  | 0.66  (Mann-Whitney U-test) |
| I | 4 | 2 |  |
| II | 5 | 0 |  |
| III | 3 | 4 |  |
| IV | 18 | 5 |  |
| Volume (cm^3^, min-max (median)) | 0.96-182.64 (18.30) | 1.91-325.59 (42.32) | 0.19 (Mann-Whitney U-test) |
| Smoking status |  |  | 4.70 × 10^−4^  (Mann-Whitney U-test) |
| Non-smoker | 22 | 2 |  |
| Former-smoker | 5 | 2 |  |
| Current-smoker | 3 | 7 |  |
| Ethnicity |  |  |  |
| Asian | 30 | 11 | 1.00 (Chi-squared test) |
