# Supplementary material for: Robust radiogenomics approach to the identification of EGFR mutations among patients with NSCLC from three different countries using topologically invariant Betti numbers
Source: PLoS One. 2021 Jan 11;16(1):e0244354. doi: 10.1371/journal.pone.0244354 (PMC7799813; doi:10.1371/journal.pone.0244354)
Supplement: S3 Table — (DOCX) [file pone.0244354.s003.docx]

**S3 Table. Distributions and significant differences in demographic/clinical characteristics between patients with sensitizing epidermal growth factor receptor (*EGFR*) mutants and wildtypes in a dataset obtained from The Cancer Imaging Archive.**

|  | *EGFR* mutant | *EGFR* wildtype | p value (testing method) |
| --- | --- | --- | --- |
| Total number of cases | 9 | 45 |  |
| Age (y, min-max (median)) | 55-87 (71) | 43-86 (69) | 0.40 (Mann-Whitney U-test) |
| Sex |  |  | 0.78  (Chi-squared test) |
| Male | 3 | 10 |  |
| Female | 6 | 35 |  |
| Stage |  |  | 0.02  (Mann-Whitney U-test) |
| I | 8 | 20 |  |
| II | 1 | 12 |  |
| III | 0 | 11 |  |
| IV | 0 | 2 |  |
| Volume (cm^3^, min-max (median)) | 3.16-31.25 (14.53) | 0.81-371.54 (12.46) | 0.80 (Mann-Whitney U-test) |
| Smoking status |  |  | 0.14  (Mann-Whitney U-test) |
| Non-smoker | 1 | 4 |  |
| Former-smoker | 8 | 29 |  |
| Current-smoker | 0 | 12 |  |
| Ethnicity |  |  |  |
| Caucasian | 8 | 43 | 0.40  (Chi-squared test) |
| Hispanic/Latino | 1 | 1 |  |
| Native Hawaiian/  Pacific Islander | 0 | 1 |  |
