# Supplementary material for: Robust radiogenomics approach to the identification of EGFR mutations among patients with NSCLC from three different countries using topologically invariant Betti numbers
Source: PLoS One. 2021 Jan 11;16(1):e0244354. doi: 10.1371/journal.pone.0244354 (PMC7799813; doi:10.1371/journal.pone.0244354)
Supplement: S4 Table — (DOCX) [file pone.0244354.s004.docx]

| **S4 Table. Case numbers obtained from The Cancer Imaging Archive for constructing a test dataset.** | | | | | |
| --- | --- | --- | --- | --- | --- |
| R01-007 | R01-046 | R01-065 | R01-084 | R01-106 | R01-137 |
| R01-015 | R01-047 | R01-068 | R01-086 | R01-112 | R01-139 |
| R01-020 | R01-048 | R01-070 | R01-088 | R01-117 | R01-141 |
| R01-027 | R01-051 | R01-071 | R01-090 | R01-119 | R01-142 |
| R01-028 | R01-054 | R01-074 | R01-091 | R01-122 |  |
| R01-030 | R01-056 | R01-077 | R01-094 | R01-125 |  |
| R01-035 | R01-057 | R01-078 | R01-096 | R01-129 |  |
| R01-036 | R01-062 | R01-079 | R01-097 | R01-130 |  |
| R01-040 | R01-063 | R01-080 | R01-104 | R01-132 |  |
| R01-045 | R01-064 | R01-082 | R01-105 | R01-133 |  |
