# Supplementary material for: Robust radiogenomics approach to the identification of EGFR mutations among patients with NSCLC from three different countries using topologically invariant Betti numbers
Source: PLoS One. 2021 Jan 11;16(1):e0244354. doi: 10.1371/journal.pone.0244354 (PMC7799813; doi:10.1371/journal.pone.0244354)
Supplement: S5 Table — (DOCX) [file pone.0244354.s005.docx]

**S5 Table. Case numbers selected from The Cancer Imaging Archive for constructing a multi segmentation dataset.**

| LIDC-IDRI | QIN LUNG CT | RIDER Lung CT |
| --- | --- | --- |
| LIDC-IDRI-0314  LIDC-IDRI-0325  LIDC-IDRI-0580  LIDC-IDRI-0766  LIDC-IDRI-0771  LIDC-IDRI-0811  LIDC-IDRI-0905  LIDC-IDRI-0963  LIDC-IDRI-0965  LIDC-IDRI-1012 | QIN-LSC-0003  QIN-LSC-0009  QIN-LSC-0014  QIN-LSC-0028  QIN-LSC-0049  QIN-LSC-0055  QIN-LSC-0064  QIN-LSC-0088  QIN-LUNG-01-0007  QIN-LUNG-01-0013 | RIDER-1129164940  RIDER-1332496276  RIDER-1500037140  RIDER-1825099523  RIDER-2151469008  RIDER-2283289298  RIDER-2541949645  RIDER-2655999012  RIDER-2799584460  RIDER-3023568408 |
