# Supplementary material for: Robust radiogenomics approach to the identification of EGFR mutations among patients with NSCLC from three different countries using topologically invariant Betti numbers
Source: PLoS One. 2021 Jan 11;16(1):e0244354. doi: 10.1371/journal.pone.0244354 (PMC7799813; doi:10.1371/journal.pone.0244354)
Supplement: S6 Table — (DOCX) [file pone.0244354.s006.docx]

| **S6 Table. Radiomic features with the feature types.** | | | | |
| --- | --- | --- | --- | --- |
| Histogram-based features (n = 14) | Texture features (n = 40) | | | |
|  | GLCM (n = 9) [1] | GLRLM (n = 13) [2] | GLSZM (n = 13) [3] | NGTDM (n = 5) [4] |
| Energy  Entropy  Kurtosis  Maximum  Mean  Mean absolute difference  Median  Minimum  Range  Root mean square  Skewness  Standard deviation (SD)  Uniformity  Variance | Energy  Contrast  Entropy  Homogeneity  Correlation  Variance  Sum average  Dissimilarity  Auto correlation | Short run emphasis (SRE)  Long run emphasis (LRE)  Gray level non-uniformity (GLN)  Run length non-uniformity (RLN)  Run percentage (RP)  Low gray level run emphasis (LGRE)  High gray level run emphasis (HGRE)  Short run low gray level emphasis (SRLGE)  Short run high gray level emphasis (SRHGE)  Long run low gray level emphasis (LRLGE)  Long run high gray level emphasis (LRHGE)  Gray level variance (GLV)  Run-length variance (RLV) | Small zone emphasis (SZE)  Large zone emphasis (LZE)  Gray level non-uniformity (GLN)  Zone-size non-uniformity (ZSN)  Zone percentage (ZP)  Low gray level zone emphasis (LGZE)  High gray level zone emphasis (HGZE)  Small zone low gray level emphasis (SZLGE)  Small zone high gray level emphasis (SZHGE)  Large zone low gray level emphasis (LZLGE)  Large zone high gray level emphasis (LZHGE)  Gray level variance (GLV)  Zone-size variance (ZSV) | Coarseness  Contrast  Busyness  Complexity  Strength |

GLCM: Gray level Co-occurrence Matrix; GLRLM: Gray level Run-Length Matrix; GLSZM: Gray level Size Zone Matrix; NGTDM: Neighborhood Gray-Tone Difference Matrix

1. Haralick RM, Shanmugam K, Dinstein I. Textural features for image classification. *IEEE Trans Syst Man Cybern*. 1973;3:610–21. doi:10.1109/TSMC.1973.4309314.
2. Galloway MM. Texture analysis using gray level run lengths. *Comput Graph Image Process.* 1975;4:172–9. doi:10.1016/S0146-664X(75)80008-6.
3. Thibault, G. Texture indexes and gray level size zone matrix. Application to cell nuclei classification. *In Proc. Pattern Recognition and Information Processing*. Minsk, Belarus, 140−145, 2009.
4. Amadasun M, King R. Textural features corresponding to textural properties. *IEEE Trans Syst Man Cybern*. 1989;19:1264−1274. doi:10.1109/21.44046.
